# Timing between age at first sexual intercourse and age at first use of contraception among adolescents and young adults in Niger: What role do education and place of residence play?

**DOI:** 10.12688/gatesopenres.12972.1

**Published:** 2019-05-10

**Authors:** Abibatou Agbéké Olakunle, Boladé Hamed Banougnin

**Affiliations:** 1 Department of Statistics, University of Ibadan, Ibadan, Oyo, Nigeria; 2 Pan African University, Life and Earth Science Institute (Including Health and Agriculture), University of Ibadan, Ibadan, Oyo, Nigeria

**Keywords:** Education, First sexual intercourse, Niger, place of residence, survival analysis, uptake of modern contraceptive, women aged 15-24

## Abstract

**Background: **Low contraceptive use among women in Niger is one of main causes of early childbearing and unwanted pregnancies, which affect maternal and child health. Education and place of residence have been cited as factors affecting modern contraceptive use.

**Methods: **We investigated the separate and joint effects of the place of residence and education on the time to modern contraceptive uptake among women aged 15-24 in Niger. The study used data from the second round of the 2016 Niger Performance Monitoring and Accountability 2020 (PMA2020) project. Survival analysis was applied for 830 women.

**Results: **Nelson-Aalen curves show that urban women had higher hazards of (and shorter delays in) modern contraceptive uptake as compared to their rural counterparts. Also, the higher the level of education, the higher the hazards of (and the shorter the delays in) modern contraceptive uptake. Findings from the multivariate (survival) analysis confirms these figures and provides the net effect of the place of residence on modern contraceptive uptake. Whether living in urban or rural areas of Niger, what matters more is the level of education.

**Conclusions**: Family planning programmes concerning adolescent and young women should focus more on women with no education and those that are illiterate.

## Introduction

Niger presents worrying characteristics for youth sexual reproductive health. The country has the highest fertility rate as well as the lowest age for marriage and childbearing in Africa and the world ( Barroy *et al.*, 2015). With a total fertility rate of 7.6 children per women and a population growth rate of 3.9%, Niger’s population has increased from 7,292,000 in 1989 to 21,311,000 in 2018 ( United Nations, 2019). Early marriage and, especially, childbearing are the main causes driving the high population growth and fertility in Niger ( Nouhou, 2016). In general, early marriage and childbearing is also associated with increased risk of maternal death and new-born death ( Gibbs *et al.*, 2012). The 2018 Niger Performance Monitoring and Accountability 2020 (PMA2020) family planning brief reports that more than 70.9% of women aged 18–24 were married by age 18 ( INS - Niger, 2018). The brief also reports that one-third of Niger women aged 18–24 had their first birth by age 18. Moreover, estimates from UNICEF (2008) show that a woman’s lifetime risk of dying due to complications caused by pregnancy or childbirth was one in seven. This represents 14,000 deaths among Nigerien mothers from pregnancy-related causes. The maternal mortality rate in Niger was 553 per 100,000 live births in 2015 ( Roser & Ritchie, 2019b) and the under-five mortality rate was 84.5 deaths per 1,000 live births in 2017 ( Roser, 2019a). These figures reflect the poor health of both mother and child in Niger.

One of the major causes of maternal and childhood mortality remains the early age at first birth which typically depends on the age at first sexual intercourse. Evidence has shown that there is an inverse association between age at first sexual intercourse and early childbearing ( Gebreselassie & Govindasamy, 2013; Khatiwada *et al.*, 2013; Meekers, 1993; Westoff, 2003). Data from the 2012 Niger Multiple Indicator Cluster Survey (MICS) and Demographic and Health Survey (DHS) show that 28% of women had already had their first sexual intercourse by age 15, and 45% had given birth by age 18 ( INS - Niger *et al.*, 2013). These figures are among the highest in sub-Saharan Africa ( ICF International, 2018). This is an age at which adolescents are most susceptible to sexually transmitted infections, including HIV/AIDS and human papillomavirus and other health complications ( Fagbamigbe *et al.*, 2015). The modern contraceptive is one of the means of promoting good reproductive health, especially among adolescents and young women.

Sub-Saharan Africa has one of the highest rates of teenage pregnancy ( Yakubu & Salisu, 2018) and the lowest contraceptive prevalence rates ( Radovich *et al.*, 2018) in the world. The low contraceptive use among Niger adolescents (4%) and youths (14%) ( ICF International, 2018) is one of the main causes of early childbearing and unwanted pregnancies, which have many consequences including unsafe abortion, high maternal and child mortality and reduced earning potential and educational achievements. This calls for an increasing interest in expanding contraceptive use among adolescents and youths as advocated by the Family Planning 2020 global partnership ( Family Planning 2020, 2015).

The research literature has shown that the sooner the use of contraceptive methods, especially modern methods (pill, intrauterine device, injectables, female condom, male condom, female sterilization, male sterilization, implants and lactational amenorrhea), the higher the odds of preventing early childbirth ( Abma & Martinez, 2017; Guleria *et al.*, 2017; Shu *et al.*, 2016). Many factors affecting the use of contraceptive methods in the sub-Sahara African context have been observed. Many of them repeatedly cited women’s level of education and place of residence as major determinants of contraceptive use ( Fawole & Adeoye, 2015; Nair & Devi, 2017; Ochako *et al.*, 2017). Most of these studies have examined the separated effect of the place of residence and education on contraceptive use. Likewise, few studies have been carried out on factors affecting the timing between first sexual intercourse and first use of contraceptive methods. Furthermore, the third target of Sustainable Development Goals (SDGs) aims to reduce maternal mortality and under-five mortality to less than 70 deaths per 100,000 live births and 25 deaths per 1,000 live births ( Gostin & Friedman, 2015). There is an expectation that through the use of modern contraceptive methods (from the very first sexual initiation), there will be an improvement in maternal and child health in Niger. This study aims to investigate both separated and joint effects of the place of residence and education on the timing between first sexual intercourse and first use of modern methods of contraception.

## Methods

### Data

The study draws on data from the Performance Monitoring and Accountability 2020 (PMA2020) project. PMA2020 supports low-cost, rapid-turnaround survey monitoring key indicators for family planning, water, sanitation and hygiene, and other health and development indicators in 11 low to middle income countries (for more details concerning the methods and objectives of PMA2020, see Johns Hopkins University, 2013). The PMA2020 project received approval to collect data from Institutional Review Boards at Johns Hopkins Bloomberg School of Public Health and the Niger Institute of Statistics.

We use data from the second round of Niger PMA2020 survey conducted from February to April 2016 ( Performance Monitoring and Accountability 2020 Project, 2016). The 2016 Niger PMA2020 survey used a two-stage cluster sample design with Niamey (the capital city of Niger), urban areas outside of Niamey, and rural areas as strata. This sample design enabled national and sub-national estimates to be produced. The study selected 84 enumeration areas (EAs) based on the 2012 Niger census’ sample frame, and randomly selected 35 households within each EA. Then, all resident women of reproductive ages within the sampled households were interviewed. The final dataset included 2,785 households (96.2%) and 3,048 women of reproductive ages (95.5%).

Our study population consisted of 1,238 women aged 15–24 at the time of the survey. Several studies in the areas of sexual and reproductive health have defined young women as those falling within the ages of 15–24 years ( Ali & Cleland, 2005; Dellar *et al.*, 2015; Gouws *et al.*, 2008; Lauby & Stark, 1988). Furthermore, young women undergo significant transitions in lifestyle, maturity, and legal rights between these ages; which places them at different vulnerabilities at different time points ( Dellar *et al.*, 2015). Of the 1,238 women aged 15–24, 749 (60.5%) had already had their first sexual intercourse. Among these 749 women, we excluded 31 who reported having their first sexual intercourse before age 4, 26 who did not know when they first had sex, and 18 missing values. This resulted in a sample of 674 young women. From these, 671 were able to provide information on whether and, if so, when they have used a modern method of contraception for the first time. The corresponding weighted number of women aged 15–24 who had ever had sex is 830 ^1^ (69.6% of women aged 15–24). None of the remaining 567 women (who had never had sex or did not give any consistent answer about the experience of the first sexual intercourse) use contraception.

### Measures

The 2016 Niger PMA2020 survey questionnaire was extensive and included sections on reproduction, pregnancy and fertility preferences, contraception, sexual activity, menstrual hygiene, and location. In particular, the questionnaire provided data on family planning use, of the type gathered in the Demographic and Health Surveys. Throughout this study, pill, intrauterine device, injectables, female condom, male condom, female sterilization, male sterilization, implants and lactational amenorrhea are referred as modern methods of contraception. The survey also collected information on the respondent’s socioeconomic characteristics, including educational attainment, marital status, and location.

The dependent variable of the study is the time to uptake of a modern contraceptive use since the first sexual intercourse. The time to uptake of a modern contraceptive use is calculated in years and is equal to the age at first modern contraceptive use minus the age at first sexual intercourse for modern contraceptive users. For non-users, the time to uptake of a modern contraceptive use is equal to the current age minus the age at first sexual intercourse. PMA2020 survey asks four questions to determine the time to uptake of a modern use: “How old were you when you first had sexual intercourse?”, “Have you ever done anything or tried in any way to delay or avoid getting pregnant?”, “How old were you when you first used a method to delay or avoid getting pregnant?”, and “Which method did you first use to delay or avoid getting pregnant?”.

The main independent variables are the place of residence and education. During the 2016 Niger PMA2020 survey, women were asked the following question about their educational attainment: “What is the highest level of school you attended?”. The possible response categories to this question were: ‘Never attended’, ‘Primary’, ‘Secondary’, and ‘Higher’. Only a very small proportion (0.4%) of young women had a higher level of education. To prevent non-accurate p-value for the test analysis, we combined the ‘Secondary’ and ‘Higher education’ categories into one (‘Secondary or higher’). The place of residence is divided into two categories: urban and rural areas. As mentioned above, the odds of modern contraceptive use usually depend (non-exhaustively) on the availability by location (urban/rural residence) and knowledge (education) ( Kandala *et al.*, 2015; Kravdal, 2002; Rutenberg *et al.*, 1991). In addition to education and place of residence, other variables (of control) such as age and household wealth tertile. Empirical data point out wealth as affordability factor, and age as confidence-to-buy factor affecting differences in modern contraceptive use across social groups ( O’Regan & Thompson, 2017; Wambui *et al.*, 2009).

### Analytic methods

We used survival analysis since the main outcome of this study (time to modern contraceptive uptake) is a form of time to event. The survival time is assumed to begin when a woman has her first sexual intercourse until when she starts using modern contraceptives. Young women who have never used any modern contraceptive at the time of interview are right censored as of the date of the survey. Let *T* be a non-negative random variable representing the duration from first sexual intercourse to modern contraceptive uptake. The subjects at risk are the 671 (830 weighted) women aged 15–24, followed-up since their first sexual intercourse. The observation continues until time *t*. If a woman has taken up modern contraceptive, the time *t* is the time of modern contraceptive uptake; otherwise, the time *t* is the time of the survey (in 2016). The observation ends for a woman at time *T* = *t* if she has started using any modern contraceptive method. For example, *T* = 0 if a woman started using modern contraception before one year after her first sexual intercourse. Assuming that *T* is a continuous random variable with probability density function *f*( *t*), the cumulative distribution function (c.d.f), giving the probability that a woman has taken up modern contraceptive by duration *t* is:

$$F(t)=Prob(T\leq t)=\int_{0}^{t}f(s)ds\;(1)$$

The complement of the c.d.f ( *S*( *t*) = 1 – *F*( *t*)), which represents the probability that a woman is yet to take up modern contraceptive by duration *t*, is the survival function. In this study, we defined the survival function in terms of the hazard function, which is the instantaneous rate of occurrence of the event of interest (here, the uptake of modern contraceptive since first sexual intercourse). The hazard function is defined as:

$$h(t){=}_{\;dt\to 0}^{\;\lim}\frac{Prob(t\;<\;T\;<\;t\;+\;dt|T\;>\;t)}{dt}\;(2)$$

The numerator of this expression is the conditional probability that a woman takes up modern contraceptive in the interval [ *t,t + dt*) given that she has not used modern contraceptive before. The denominator is the width of the interval. Dividing one by the other, we obtain a rate of uptake of modern contraceptive per unit of time (i.e. per year). Taking the limit as the width of the interval goes down to zero, we obtain an instantaneous rate of uptake of modern contraceptive.

The survival analysis for this paper proceeded in two steps. First, we used Nelson-Aalen nonparametric estimates of cumulative hazard rate function for the descriptive analysis. Especially, we drew cumulative hazard rate curves of time to modern contraceptive uptake for all women aged 15–24, and by groups (stratifying by education and place of residence). We used log-rank tests to test the equality of the cumulative hazard rate functions across groups. Second, we employed (multivariate) Cox semi-parametric proportional-hazards models to predict how the place of residence and education affect the time to uptake of modern contraceptive. The hazard rate in a Cox proportional-hazards model is defined as:

$$h(t|x,\beta )=h_{0}(t)\;\exp(x^{\prime }\;\beta )\;(3)$$

where *x* is the vector of independent variables (education, place of residence, age, and wealth tertile), *h*_0_( *t*) is the baseline hazard function, and *β* is the vector of coefficients. The multivariate analysis consisted of three different models. All models were controlled for age and wealth tertile. The first model (Model 1) presents the effect of the place of residence on the time to uptake of modern contraceptive use. The second model (Model 2) is equal to Model 1 plus the education variable. The third model (Model 3) shows how the interaction between the place and residence and education influences the timing to modern contraceptive uptake. We applied sampling weights for the analysis. Stata 13 was used to analyse the data.

## Results

We first report descriptive statistics of the variables used in the analysis in Table 1. Mean age at the first sexual intercourse among women aged 15–24 was 19.7 years. Just under one-quarter (197 out of 830; 23.7%) of the respondents (ever-sexually-active women aged 15–24) had used contraception. Over three-quarters of (first-time) contraceptive users (156 out of 197) had practiced modern methods. The pill is the most widely used modern method, accounting for more than three-fifths of modern contraceptive use and 11.8% of the respondents. Of all women aged 15–24, very few (0.6%) had used condoms for their first contraceptive experience. Table 1 also indicates that the vast majority of the respondents (86.4%) were living in rural areas. At the time of the survey, only 12.2% of ever-sexually-active women aged 15–24 had attained secondary or higher level of education, whereas more than two-thirds (68.3%) had never attended school. The mean time between first sexual intercourse and first use of modern contraceptive was 3 years with a standard deviation of 2.1 years.

**Table 1.** Distribution of factors concerning contraceptive use and demographic variables.

Variable *	Number of weighted observations = 830		
	Percentage	Mean	Std. Dev.
Contraceptive use			
Modern methods	18.7		
Implants	1.8		
Injectables	4.6		
Pill	11.8		
Male condoms	0.6		
Traditional	4.9		
Lactational Amenorrhea Method (LAM)	0.2		
Rhythm	0.1		
Withdrawal	0.1		
Other traditional	4.6		
Total users	23.7		
Nonusers	75.3		
Unknown	1.0		
Place of residence			
Urban	13.6		
Rural	86.4		
Education			
No education	68.3		
Primary	19.5		
Secondary or higher	12.2		
Wealth tertile			
Poorest	33.0		
Middle	31.9		
Richest	35.1		
Age, years		19.7	2.3
Time to modern contraceptive uptake, years ^(a)^		3.0	2.1
Time between first sex and interview date, years ^(b)^		4.4	2.7
Total	100.0		

*No variable contains missing value; ^(a)^ For women who have ever used modern contraceptive (18.7%); ^(b)^ For women who have never used modern contraceptive (75.3%). Std. Dev., standard deviation.

**Figure 1.**
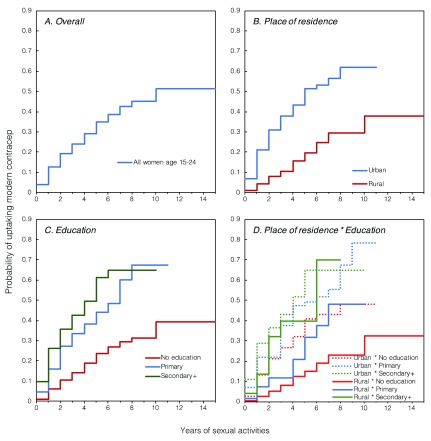
Nelson-Aalen curve showing the cumulative hazard of modern contraceptive uptake among women aged 15–24 in Niger, according to the place of residence and education.

We then compared the incidence rate of modern contraceptive uptake among ever-sexually-active young women by place of residence ( Figure 1B) and education ( Figure 1C). Figure 1B reveals two important findings. First, the incidence rate of modern contraceptive uptake increases very fast in the first six years after sexual initiation. Between six and eight years post sexual initiation, the incidence rate continues to rise steadily though slower than the first six years. Secondly, the incidence rate of modern contraceptive uptake since sexual initiation was higher among urban women than rural women. In addition, the cumulative hazard curve of modern contraceptive uptake reached its peak faster among urban women (61.8% at the tenth year) than rural women (37.8% at the fourteenth year). As expected, the incidence rate of modern contraceptive uptake is higher among women with higher education than those with no education ( Figure 1C). Also, the higher the educational level, the faster the modern contraceptive uptake. Figure 1D shows how interaction between place of residence and education is associated with the chances of modern contraceptive uptake. Urban women with primary or higher education and rural women secondary or higher education had the highest incidences of and shorter delays in modern contraceptive uptake. The Log-rank test for equality of survival functions shows significant differences in the cumulative hazard function of place of residence and education.

Table 2 presents the results of Cox semi-parametric proportional-hazards models of the effects of the place of residence and education on time to modern contraceptive uptake. We fitted three models for the survival data. Model 1 was restricted to place of residence (with wealth and age as control variables). Significant differences are observed between urban and rural women. The probability of modern contraceptive uptake was 1.73 (95% CI: 1.09–2.73) times higher for urban women as compared with rural women. According to the wealth status, women in the richest wealth tertile were more than twice as likely to take up modern contraceptive than women in the poorest wealth tertile. The chances of modern contraceptive uptake increased by 11% per one-year increase in age, all other factors being equal.

In Model 2, we introduced education, which was not included in Model 1. There was no significant difference in modern contraceptive uptake between urban and rural women after including education. Findings from Model 2 in Table 2 show that the higher the education the higher the hazard of modern contraceptive initiation. The hazard ratio (HR) of modern contraceptive uptake was higher among women with primary education (HR = 1.80, *p* < 0.01, 95% CI: 1.20–2.72) and women with at least secondary education (HR = 2.30, *p* < 0.01, 95% CI: 1.43–3.70) compared with their peers with no education.

**Table 2.** Relative risks (hazard ratios) of modern contraceptive uptake among women aged 15–24 in Niger.

Variable	Model 1		Model 2		Model 3	
	HR	(95% CI)	HR	(95% CI)	HR	(95% CI)
Place of residence						
Urban	1.73 **	(1.09–2.73)	1.46	(0.92–2.33)		
Rural	1		1			
Education						
No education			1			
Primary			1.80 ***	(1.20–2.72)		
Secondary or higher			2.30 ***	(1.43–3.70)		
Place of residence * Education						
Urban * No education					1.72	(0.87–3.40)
Urban * Primary					3.17 ***	(1.54–6.53)
Urban * Secondary or higher					2.87 ***	(1.42–5.79)
Rural * No education					1	
Rural * Primary					1.74 **	(1.08–2.79)
Rural * Secondary or higher					2.82 ***	(1.60–4.96)
Wealth tertile						
Poorest	1		1		1	
Middle	1.46	(0.91–2.33)	1.52 *	(0.95–2.44)	1.50 *	(0.94–2.40)
Richest	2.18 ***	(1.37–3.49)	1.99 ***	(1.23–3.22)	1.88 **	(1.15–3.08)
Age	1.11 **	(1.02–1.21)	1.10 **	(1.01–1.21)	1.11 **	(1.01–1.21)
Subjects	773					
Time at risk	3,409					
Failures	140					

*p<0.1; **p<0.05; ***p<0.01. HR, hazard ratio; CI, confidence interval.

Model 3 included the interaction terms between place of residence and education without the separated variables (education and place of residence). Urban women with primary education had the highest hazard of modern contraceptive uptake (HR = 3.17, *p* < 0.01, 95% CI: 1.54–6.53). They are followed by their peers with secondary or higher education (HR = 2.87, *p* < 0.01, 95% CI: 1.42–5.79) and rural women with secondary or higher education (HR = 2.82, *p* < 0.01, 95% CI: 1.60–4.96). Also, women with no education had similar hazard rates of modern contraceptive uptake whether living in urban or rural areas. In other words, the educational status is the most discriminating factor for modern contraceptive uptake.

## Discussion

Identifying investments needed to improve family planning programmes in developing countries remains a source of concern for health advocates and professionals, policies makers and funding agencies ( Sedgh *et al*., 2016). Improvement in modern contraception use should enable women—irrespective of their place of residence and socioeconomic status—to prevent reproductive health complications. This study used data from a nationally representative sample of Niger young women (aged 15–24 years) to examine how the place of residence and education, as well as their interaction, affect the time to uptake of modern contraceptives. Findings from the analysis show that residing in urban areas, having a high educational level and living in the richest wealthy tertile are associated with higher hazard rates of and shorter delays in modern contraceptive uptake. Moreover, the hazard ratio of using modern contraceptive among young women also increases with age.

Findings from multivariate (survival) analysis showed a minor effect of the place of residence on the time to modern contraceptive uptake when adjusted for education (and the main control variables: age and wealth tertile). Indeed, the difference between urban and rural women in modern contraceptive uptake become non-significant after adjusting for the level of education. Thus, the level of education seems to counterbalance the effect of increased time to uptake of modern contraceptives among rural women (ages 15–24) in Niger.

Therefore, the positive relationship between education and the hazard rates of modern contraceptive uptake confirms previous work on the association between education and contraceptive use ( Khan & Mishra, 2008; O’Regan & Thompson, 2017; Selassie, 2017). Better education is not only positively associated with early use of contraception, but also contributes to empowerment of women ( Family Planning 2020, 2014; Tadesse *et al*., 2013; Tuladhar *et al*., 2013). Also, women who attend at least a primary level of education (and those who are literate) are more likely to know about family planning services through media ( Fagbamigbe *et al*., 2015; Shapiro & Tambashe, 1994). Finally, we further our analysis by introducing interaction terms between the place of residence and education. In so doing, statistically significant differences emerged. Urban women with at least primary education and rural women with secondary or higher education have approximatively the same hazards of modern contraceptive uptake. This confirms the key role played by education in modern contraceptive uptake among young women in Niger.

## Conclusion

Our results suggest that the delay in modern contraceptive uptake is relatively high. This delay is higher among rural and less educated women than among urban and more educated women. Therefore, adolescents and youths in Niger, especially those living in rural areas and those with a low educational level, are at risk of early childbirth and its consequences. These consequences include among others, child and maternal mortality, school dropout and low participation in the labour market. In consequence, there is a need for policies and programmes that empower adolescents and youths through improving information about the access to, and utilization of reproductive health services. It is necessary to reduce all barriers to access and improve the contraceptive distribution system by integrating family planning activities into national policies on reproductive health with more focus on women with no education.

## Data availability

Underlying data used in this study were obtained from PMA2020 (Niger Round 2, 2016) ( Performance Monitoring and Accountability 2020 Project, 2016). All PMA2020 datasets are free to download and use, although users are required to register for a PMA2020 dataset account. The request form must include a brief description of the research or analysis that the user would like to conduct using the requested data. Information about the data and terms of use are available here.

## Footnotes

^1^ The PMA2020 sampling design follows DHS’ standard, and as described in https://dhsprogram.com/pubs/pdf/DHSG1/Guide_to_DHS_Statistics_DHS-7.pdf, such data must be weighted. The PMA2020 also includes the sampling weight variable. Weighting the female data, the corresponding number of observations is 830.
