# Supplementary material for: Timing between age at first sexual intercourse and age at first use of contraception among adolescents and young adults in Niger: What role do education and place of residence play?
Source: Gates Open Res. 2019 May 10;3:1463. [Version 1] doi: 10.12688/gatesopenres.12972.1 (PMC6795034; doi:10.12688/gatesopenres.12972.1)
Supplement: Peer review history

# Peer review: Reviewer response for version 1

**DOI:** 10.21956/gatesopenres.14079.r28037

**Published:** 2019-10-28

**Authors:** Michael J. Mahande

**Affiliations:** 1 Department of Epidemiology and Biostatistics, Institute of Public Health, Kilimanjaro Christian Medical University College, Moshi, Tanzania

**Section**

**Comment, question, suggestions.**

**Abstract**

1. Well-constructed clear and short abstract.
2. Easily understandable.

**Introduction**

**Background**

1. Clear background with enough information.
2. A broader explanation as to what we can’t do by not having the data on timing would be important to the readers i.e. what is the knowledge gap?

**Objectives**

1. It was better to clearly explain what was meant by separate and joint effects of residence and education.

**Methodology**

1. WHO and UNICEF have standard definitions of adolescent and youths. It would better to use standard references from the WHO/UNICEF rather than citing papers.
2. The information regarding exclusion criteria would have been easier to understand if presented using a flow chart rather than narratives.
3. Why only focus on age and residence as the adjusting variables? What about other independent variables? Such as partner information (age and education levels), Family Planning media exposure, marital status, religion etc? All these have also been shown to affect modern contraceptive use. I am concerned with the aspect of residual confounding caused by leaving these variables out from the analysis.
4. The dependent variable was measured in years. Is it complete years? What if a woman started using a modern method 3 months after her first sexual encounter? How would she be recorded?
5. Do you mean multi-variate or multi-variable analysis? Please check these as they are totally different
6. I have some few concerns with the model building process.
  1. Generally, model 1 should be an empty model containing the crude estimates of an exposure variable on the outcome, in this case a model with education alone or a model with residence alone.
  2. Model 2 continues to start adjusting for other exposure variables and estimating confounding effect when an exposure var results in a 10%- 15% change in the estimates of the primary predictor of interest.
  3. An interaction is looked at based on plausibility of its effect, for instance, do we have reason to believe that modern contraceptive use differs among urban women based on the levels of their education? Similarly, with women residing in rural areas.
  4. Finally, was model diagnostics performed for the Cox-ph models? Was the Ph assumption holding or was it violated?
7. Was the data both st and svyset?

**Results **

1. In the narratives you mention the frequencies of the variables, these are not shown in table 1. Would be better to present them as well since they are present in your narratives.
2. Would have also been interesting to show during the first sexual encounter, how many women utilized modern contraceptives?, and who are these women? (if this information is available in the dataset).
3. Why was it important to show in table 1 the time between first sexual encounter and the date of interview? What was this information telling the readers?
4. The information on median time to contraceptive use should appear earlier in the narratives since it is of much interest.
5. Please correct the y-axis in figure 1 to say cumulative hazards and not probabilities. Nelson Aalen curves are not probabilities.
6. Please indicate the log-rank p-values on the graphs in order to show the significance difference in modern contraceptive uptake.
7. Most of the interpretations are in-correct. For survival analysis cox Ph models we interpret the “Hazards”, and not probabilities/chance/likely. **Please correct this -major comment.**
8. For model 3: Why did you leave out the main effects and only included the interaction term alone? This information is necessary and important.
9. Why focus on a significance level of <0.1 rather than the tradition <0.05 for table 2? Please provide description in the methodology under data analysis section.
10. “In other words, the educational status is the most discriminating factor for modern contraceptive uptake.”
   
  1. Since this is an interaction, it indicates that modern contraceptive use among urban women differs according to their levels of education and similarly, among modern contraceptive use, rural women differs according to their levels of education.
  2. An interaction shows that the outcome among each level of an exposure variable differs across the levels of a third variable.
  3. Which means that residence has an effect on modern contraceptive use differently across education categories.
  4. Had you included the main effects in this model 3, it would also have added some information on the significance of the individual variables.

**Discussion**

 Can be better synthesized to bring out the meaning of the results and their implications to policy and practice.

**Strengths and limitations**

 Missed the section on strengths and limitations of the study.

**Conclusion + Recommendations**

 Conclusion and recommendation were focused on variables which were not assessed in the study i.e. FP media exposure, roles of empowerment of women and removing barriers to access and distribution of modern contraceptives. All these were not measured in the present study.

I confirm that I have read this submission and believe that I have an appropriate level of expertise to state that I do not consider it to be of an acceptable scientific standard, for reasons outlined above.

# Peer review: Reviewer response for version 1

**DOI:** 10.21956/gatesopenres.14079.r28038

**Published:** 2019-10-15

**Authors:** Waliu Jawula Salisu

**Affiliations:** 1 School of Nursing and Midwifery, Tehran University of Medical Sciences, Tehran, Iran

The paper sheds light on the relationship between education and place of residence concerning modern contraceptive use among young women in Niger.

 The author’s highlight on the gap in the literature however, seems to focus primarily on Sub Saharan Africa rather than the original study context. The gap in the literature that necessitates the study in Niger is unclear. A little emphasis on the literature gap in Niger is needed.

 In the results section, the following statement meant to describe Figure 1 seems misleading since it did not feature any percentages on either axis of the figures. “…the cumulative hazard curve of modern contraceptive uptake reached its peak faster among urban women (61.8% at the tenth year) than rural women (37.8% at the fourteenth year)...”. I suggest the authors take note and amend it appropriately.

I confirm that I have read this submission and believe that I have an appropriate level of expertise to confirm that it is of an acceptable scientific standard.

# Peer review: Reviewer response for version 1

**DOI:** 10.21956/gatesopenres.14079.r27393

**Published:** 2019-07-08

**Authors:** Nouhou Abdoul Moumouni

**Affiliations:** 1 Organiser l'avancement des solutions au Sahel, L'Initiative OASIS Niger, Niamey, Niger

Model 3 explains the modern contraceptive uptake by the (education)*(place of residence) interaction (controlling by wealth tertile and age). What about the marginal effects of both education and place of the residence in this model? These two factors may still have some marginal effects, something to be checked by adding them into the Model 3, along with the interaction terms (let’s call this Model 4). You could estimate this Model 4 and not to include the results in the Table 2, but rather explain and comment them in few sentences (what did you find? What are the implications). If this is not feasible because of a "small sample size" problem (preventing non-accurate p-value), it should be mentioned clearly.

 The Model 3 shows that “urban women with primary education had the HIGHEST hazard of modern contraceptive uptake (HR = 3.17, p < 0.01, 95% CI: 1.54–6.53)”. Indeed, when comparing the different sub-groups from Model 3, the Relative risks (RR) could be ranked as (Urban * Primary) > (Urban * Secondary or higher) =~ (Rural * Secondary or higher). This important result was not sufficiently discussed. According to these results, when a woman attains a secondary or higher education level, the place of residence is of no importance whatsoever when it comes to modern contraceptive uptake. On the other hand, even with a primary level, women living in urban areas have a greater chance of turning to modern contraception faster after their first sexual intercourse. This result highlights an important role of the urban environment (accessibility to methods, quality, confidentiality, etc., see PMA2020 data on the quality of FP services in Niamey and complete the discussions section accordingly).

I confirm that I have read this submission and believe that I have an appropriate level of expertise to confirm that it is of an acceptable scientific standard.

# Peer review: Reviewer response for version 1

**DOI:** 10.21956/gatesopenres.14079.r27169

**Published:** 2019-07-01

**Authors:** David Lindstrom

**Affiliations:** 1 Population Studies Centre, Brown University, Providence, RI, USA

The paper addresses an important topic and the analysis is reasonably constructed, but there are a number of problems with the presentation and interpretation of the results.

1. The purpose of the paper, which is stated at the end of the last paragraph before the Methods section, should appear sooner in the paper. Perhaps, at the end of the first paragraph.
2. For the table with the descriptive statistics, the number of observations with weights should be the same as the unweighted number of observations. Multiplying the weights by the ratio of the unweighted number of observations and the weighted number of observations will preserve the relative differences in the weights and not change the total n.
3. Figure 1 presents cumulative hazards. Cumulative hazards are not probabilities. The vertical axis title should be changed to cumulative hazard. In the text the authors interpret the cumulative hazards as percents - this is not correct). 
4. The log rank test is a test of the null hypothesis of no group differences. It is not a test of comparisons between groups when there are more than 2 groups. Rejecting the null hypothesis when there are 3 or more groups means that at least one group is different from the others.
5. The authors interpret the hazard ratios in Table 2 in terms of probabilities. Strictly speaking, the hazard ratio compares two hazards, which are instantaneous failure rates and not necessarily probabilities.
6. The authors find that place of residence is not significant when education is included in the model. Why then are interactions between place of residence and education tested?
7. A proper test of the interactions between place of residence and education would also include the main effects in the model. Including the main effects provides a clearer test of whether there are indeed any significant differences in the effects of education by place of residence than a model that only includes interaction terms.

I confirm that I have read this submission and believe that I have an appropriate level of expertise to confirm that it is of an acceptable scientific standard, however I have significant reservations, as outlined above.
